# Meeting Report: Harmonization of RSV therapeutics – from design to performance

**DOI:** 10.7189/jogh.06.010205

**Published:** 2016-06

**Authors:** Harish Nair, Octavio Ramilo, Irmgard Eichler, Eric Pelfrene, Asuncion Mejias, Fernando P Polack, Koen B. Pouwels, Joanne M. Langley, Marta Nunes, Nicoline van der Maas, Leyla Kragten–Tabatabaie, Eugenio Baraldi, Terho Heikkinen, Brigitte Fauroux, Mike Sharland, Cyrus Park, Paolo Manzoni, Nikolaos G. Papadopoulos, Federico Martinón–Torres, Renato Stein, Louis Bont, in collaboration with Respiratory Syncytial Virus Network (ReSViNET)

**Affiliations:** 1 Center for Global Health Research, Usher Institute of Population Health Sciences and Informatics, University of Edinburgh Medical School, Edinburgh, UK; 2 Paediatric Infectious Diseases, Nationwide Children’s Hospital, and The Ohio State University, Columbus, Ohio, United States of America; 3 European Medicines Agency, London, UK; 4 Fundacion INFANT, Buenos Aires, Argentina; 5 Department of Pediatrics, Vanderbilt University Medical Center, Nashville, Tennessee, USA; 6 Modelling & Economics Unit, Public Health England, London, UK; 7 Unit of PharmacoEpidemiology & PharmacoEconomics, University of Groningen, the Netherlands; 8 Canadian Center for Vaccinology, Dalhousie University, IWK Health Centre and Nova Scotia Health Authority, Halifax, Canada; 9 Department of Science and Technology/National Research Foundation: Vaccine Preventable Diseases & Medical Research Council: Respiratory and Meningeal Pathogens Research Unit, University of the Witwatersrand, Johannesburg, South Africa; 10 Department Epidemiology and Surveillance of the National Immunisation Programme, CIb–RIVM, the Netherlands; 11 Julius Clinical, Zeist, the Netherlands; 12 Women’s and Children’s Health Department, Unit of Respiratory Medicine and Allergy, Padova, Italy; 13 Department of Pediatrics, University of Turku and Turku University Hospital, Turku, Finland; 14 Noninvasive ventilation and Sleep Unit, Necker Pediatric University Hospital, Paris Descartes University, Paris, France; 15 Paediatric Infectious Diseases Research Group, St George’s University London, UK; 16 Neonatology and Neonatal Intensive Care Unit, S Anna Hospital, Torino, Italy; 17 University of Manchester, Manchester, UK; 18 Allergy Dept 2nd Pediatric Clinic, University of Athens, Athens, Greece; 19 Translational Pediatrics and Infectious Diseases, Pediatrics Department, Hóspital Clínico Universitario de Santiago de Compostela, University of Santiago, La Coruña; 20 Pediatric Pulmonology Unit, Pontifícia Universidade Católica RS, Porto Alegre, Brazil; 21 Wilhelmina Children’s Hospital, University Medical Center Utrecht, Utrecht, Netherlands

RSV is a major cause of morbidity and mortality worldwide. Although no treatment or vaccine currently exists, RSV therapeutics and preventative strategies are being evaluated in clinical trials, including phase 3 trials. Despite great prospects, the regulatory pathways of novel RSV therapeutics have been defined insufficiently. Here we report the results from the ReSViNET 2nd High–level expert meeting 2016 on RSV therapeutics, which was held in Zeist, the Netherlands on March 2nd and 3rd. This meeting was organized to advance discussion on regulatory pathways, clinical development, clinical trials, and health technology models in the RSV therapeutics field. During this meeting regulators, public health specialists, academia, non–governmental organizations and pharmaceutical companies openly discussed and addressed the needs for the successful development of RSV therapeutics and prophylaxis.

## THE NEED AND THE OPPORTUNITY

RSV infection is one of the leading causes of acute lower respiratory infection (ALRI) related hospitalization and mortality during early childhood [1]. The current burden estimates are based on limited data. To overcome this data gap and to inform policy for introduction of RSV vaccine (which appears likely in the next 5–7 years), the Bill and Melinda Gates Foundation (BMGF) funded the RSV Global Epidemiology Network (RSV GEN), a platform to bring together RSV researchers from low and middle income countries to share unpublished data from ongoing / recently completed studies. This network has contributed data from more than 75 sites for the revised RSV burden estimates for 2015 which were presented at the meeting by *Dr Nair*. It is anticipated that these results which are of huge interest to clinicians, donor agencies and policy makers will be published soon.

The 2016 ReSViNET meeting aimed to discuss the development of different RSV therapeutics, the existing hurdles and strategies to overcome these barriers. *Dr Ramilo* provided an overview of the different target populations for therapy against RSV–infection. Current therapies are limited to specific populations. Prophylaxis with anti–RSV monoclonal antibodies is directed to high–risk populations only including preterm infants, children with bronchopulmonary dysplasia and those with congenital heart disease (CHD), as well as selected children with cystic fibrosis (CF) and immunocompromised conditions. However, the great majority of patients with RSV infection including children hospitalized with severe lower tract infection, and those with mild disease managed as outpatients are treated symptomatically. Not much is known yet about the burden of RSV disease among the elderly and in individuals with Chronic Obstructive Pulmonary Disease (COPD) and there are no RSV–specific therapies for these individuals. Ramilo gave a review of vaccines in development, mentioning the pros and cons of the different vaccines strategies and he also introduced antivirals and monoclonal antibodies (Mabs) in clinical development [2]. In the presentation, the need to optimize the design of clinical studies aimed at developing both treatment and preventive strategies against RSV was discussed. He reviewed how to best select the patient populations and the clinical context to evaluate the different interventions, to define clinical endpoints, and how to put into practice the lessons that can be learned from the development of antiviral therapy for HIV or from the vaccines against pneumococcus. For defining and adapting clinical endpoints, Ramilo outlined challenges as well as opportunities and emphasized the importance of obtaining robust and comprehensive clinical data, because society wants the interventions to be cost–effective. Finally, Ramilo showed the importance of selecting laboratory markers, virologic as well as immune markers and encouraged the participants to incorporate collection of clinical samples in their clinical trials to facilitate laboratory analyses that permit a better understanding of how interventions work (or not) against RSV infection.

## REGULATORY CONSIDERATIONS IN INITIATING PAEDIATRIC CLINICAL TRIALS FOR RSV THERAPEUTICS

### Overview of pediatric development plans evaluated by Paediatric Committee PDCO: regulatory considerations for initiating pediatric trials

*Dr Eichler* gave an overview of Paediatric Investigation Plans (PIPs) for RSV–antivirals and monoclonal antibodies for which the information is available in the public domain. Main challenges for the PDCO of the European Medicines Agency (EMA), responsible for assessing the content of Paediatric investigation plans, are the current lack of generally agreed recommendations by the scientific community regarding when to best initiate antiviral treatment, and the lack of validated and agreed clinically meaningful outcome measures for evaluating the effect of RSV antivirals.

Upper respiratory tract infections caused by RSV are not considered a major clinical problem, necessitating antiviral treatment. However, in children at high risk for severe lower respiratory disease (LRD) caused by RSV, the initiation of antiviral treatment early in course of RSV infection, ie, before major tissue injury in the lower airways has occurred, could benefit them to prevent severe LRD. At present, clear definitions for bronchiolitis and/or severe LRD are lacking.

The highest disease burden is considered in the first 2 years of life; consequently, this age cohort should be included in clinical trials [3]. It would be desirable to also evaluate the efficacy and safety of antivirals in older children at high risk to develop serious RSV LRD, ie, children with chronic underlying conditions, such as immunodeficiency or neuromuscular disease. As these populations are very heterogeneous and small, the conduct of dedicated clinical studies in these populations is most likely not feasible. At present, it is unknown to what extent extrapolation of efficacy is possible from young children in whom severe RSV infection manifests clinically as bronchiolitis to older children with underlying chronic diseases, in whom RSV infections manifest as RSV pneumonia. Therefore, the generation of limited clinical data in older children with chronic underlying conditions needs to be discussed.

In the absence of validated endpoints, a consensus definition of a set of core outcome measures which should be measured and reported in all clinical trials is highly warranted to allow comparability of trial results and to validate candidate endpoints.

### Regulatory aspects related to development of vaccines for RSV

*Dr Pelfrene* outlined the main regulatory considerations for maternal immunization and subsequent determination of protective efficacy in the offspring, the primary vaccination in infants as well as the desired safety database and duration of safety follow–up for both strategies. Foremost was stressed that the trial sponsor should define the intended aim of vaccination, since ultimately this will inform the labeling claim, ie, prevention of a specific clinical presentation. The case definition for RSV disease will require subjects to meet both clinical and laboratory criteria [4]. In this regard, it was emphasized that specific and sensitive assay methods for detection of RSV breakthrough cases should be employed in a standardized manner across participating study centers. The need and feasibility of a central laboratory confirmation should also be determined. Study protocols will need to define and justify the method of specimen collection (eg, nasopharyngeal aspirate or nasal swab) and provide details on sample storage and shipping conditions.

With respect to vaccination during pregnancy, it was stated that background rates of fetal demise, prematurity and congenital aberrations need to be available. Prior to conducting clinical trials in pregnant women, demonstration of safety and immunogenicity data in healthy adults (including non–pregnant women) will be necessary, as well as favorable preclinical data to be obtained on immunology and toxicology, including experiments performed in late stage pregnant animals. Exploratory trials may provide sufficient data on trans–placental transfer and persistence of maternal antibodies in the infant. In this sense, it is recognized that duration of protection may be trial setting dependent and a function of antibody titer at birth and rate of decline in the infant. Efficacy trials are expected to be broadly inclusive but there will likely be a need to stratify or to exclude sub–groups with recognized poor trans–placental transfer, such as HIV infected subjects. For the confirmatory trials, due consideration should be given to seasonality: immunization in 3rd trimester pregnancy will need to be scheduled within an appropriate time–window so that ensuing delivery coincides with the early part of the RSV season. With regard to infant immunization, it was stressed that the aim would be to elicit a strong neutralizing antibody response with a non–T helper type 2 (Th2) biased cellular immune response. Vaccine development strategy will most probably be featuring an age–de–escalation approach, and thus first be administered to RSV seropositive adults and children before progressing to RSV–naïve infants. The question of optimal timing of immunization was raised, with primary series to start as early as feasible in infancy, taking into account the inhibitory effect of maternal antibodies. In the case of vaccinating infants born to vaccinated mothers, the estimate of duration of passive protection should be known before deciding when to start active immunization. Further on, it was conveyed that the current EU general expectation regarding a pre–licensure safety database for a novel vaccine is a minimum of 3000 exposed persons to the final dose regimen of the vaccine. Though, for the vaccination of infants, it needs to be discussed what the breadth of evidence should be, to support negligible risk of disease enhancement in the RSV–naïve population.

During the discussion, it was acknowledged that the major RSV burden and mortality occurs in low–income countries. As such, it was asserted that ideally, vaccine– and therapeutic development programs need to consider a global perspective. Hence, it would be desirable that case definitions include clinical features which are easily standardized and generalizable across the different settings. EMA urged the use of the same scoring systems or scales in the trials and meeting participants were encouraged to consider scientific qualification advice for potential candidate biomarkers/outcome measures.

## MEASURING SAFETY

### Assessing the impact of anti–RSV interventions: clinical endpoints and biomarkers

It is possible to combine clinical endpoints to evaluate anti–RSV interventions with viral factors, host immune profiles and antibody responses for the diagnosis, pathogenesis and assessment of RSV disease severity [5]. *Dr Mejias* discussed that as age goes up, a decrease of antibodies against RSV are seen in the infant, acutely infected with RSV, reflecting maternal antibody transfer. Having measured neutralizing activity, they didn’t find a perfect correlation between concentration and neutralization, which needs to be understood. In fact, standardizing antibody assays and identifying a consistent antibody threshold indicative of protection still needs to be defined. However, Mejias made clear that other biomarkers such as genomics markers have a great value as predictive tools and to objectively assess disease severity. The team formed by Mejias and Ramilo found in infants hospitalized with RSV bronchiolitis significant correlations between a molecular genomic score and (1) the clinical disease severity score, (2) duration of hospitalization and (3) duration of supplemental oxygen. They are now using these tools to understand responses to therapeutic interventions, to monitor disease progression as well as to study the normal maturation of the immune system in infants. Furthermore, in collaboration with Dr Bogaert, Mejias studied the role of bacterial colonization on RSV disease severity, and noticed that nasopharyngeal bacterial colonization with specific pathogens did not appear to be a passive phenomenon. Specifically, infants with RSV infection and colonized with *S. pneumoniae* or *non–typable H. influenzae* displayed a more severe clinical phenotype and different host transcriptional profiles. Understanding the RSV–bacterial interactions in these children is of key importance when evaluating the benefit of RSV therapeutics. The challenge consists of developing composite endpoints that include clinical, virologic and laboratory parameters to monitor responses to clinical interventions.

### Enhanced RSV disease and vaccines

There are a number of new strategies for RSV vaccines, mentioning that each formulation may present individual characteristics that theoretically decrease or increase the risk for enhanced RSV disease (ERD) [6]. *Dr Polack* discussed ERD, which has been seen only in sero–negative infants and young children who were previously immunized with a formalin inactivated RSV vaccine. For many years, the consensus was that nothing but live attenuated RSV vaccines would ever be used to immunize infants. Therefore, the characterization of ERD phenotypes was of academic interest but had limited regulatory implications. Based on the outcome in mice studies and limited human data, it may be assumed that it is worthwhile to look for Th2 endpoints. However, there is a need for a consensus definition of Th2 bias and/or a consensus set of control groups for studies. ERD does not impact sero–positive children, because better antibodies precede immunization. Polack told the audience not to rely on older subjects in phase 1 studies, since there is no ERD in patients that had RSV infection before, so that would prove to be futile.

Polack concluded that the monoclonal antibodies and maternal immunization strategies are safe. For other vaccines, awareness of steric hindrance is needed and he suggested waiting between immunization and challenge, because of the danger of misunderstanding the read out. In summary, Polack said that RSV vaccines should elicit a long–lived protective antibody of high avidity for RSV protective antigens and specific cytotoxic T lymphocytes, and, neither elicit lung eosinophils, nor bias the response to Th2.

During the discussion, the participants discussed how to measure low affinity antibodies in the clinical setting, and how to assess the risk for developing ERD. How to use the information for vaccine studies was food for thought, since a perfect model of ERD is lacking. Dr Polack stressed that if ERD occurs, it probably will manifest in many individuals, as early trials had disease rates above 50%.

## RSV COST EFFECTIVENESS AND EPIDEMIOLOGY

### RSV vaccine in development: assessing the potential cost–effectiveness in high risk adult populations

The potential cost–effectiveness of RSV vaccination in high risk adults was discussed, with a main focus on the elderly population. *Dr Pouwels* noted the importance of thinking about the target population, when evaluating vaccines. The most obvious choice would be infants, given the well–established burden among infants. However, there is accumulating evidence that RSV causes a substantial burden in high–risk adults and elderly [7]. To date, two health economic studies that evaluated vaccination of the elderly against RSV have been published [8,9]. Both studies indicated that vaccination of the elderly has the potential to be cost–effective, especially among high–risk elderly. However, both studies were performed with limited data about the burden of RSV among the elderly. Moreover, one of the main drivers of the successes of several vaccination campaigns–indirect protection by reducing transmission–was not taken into account. Hence, there is a need for an updated transmission dynamic cost–effectiveness model to evaluate which vaccination strategy is most cost–effective. A recent transmission dynamic model from Kenya, which did not focus on the elderly population, concluded that vulnerable infants could be indirectly protected by annual vaccination of all school–age children [10]. Another study from the same region, estimated that it may be sufficient to vaccinate children aged 5–10 months [11]. It is clear that more models, also incorporating direct and indirect protection of the elderly, are needed. Pouwels concluded that to assess the impact and cost–effectiveness of the different strategies using transmission dynamic models, better age–group specific virological surveillance and more data on the age– and risk–group specific burden are needed.

### Collaboration to conduct research on vaccine preventable diseases in Canada–what we are learning

The Canadian Immunization Research Network (CIRN; http://cirnetwork.ca/) includes a hospital–based surveillance network (Serious Outcomes Network) which evaluates morbidity associated with vaccine–preventable infections (eg, influenza) and vaccine efficacy in adults. CIRN collaborates with the Immunization Monitoring Program ACTive (IMPACT) which conducts similar research as well monitoring adverse events following immunization, in pediatric health centers [12]. Dr Langley noted that these networks could be of interest to the RSV field for determining the burden of disease. The supportive networks within CIRN, Social Sciences and Humanities Network, Reference Laboratory Network, and Modeling and Economic Research Network actively take part in study design the studies [13]. The focus of CIRN’s work is vaccine safety, immunogenicity and effectiveness, vaccine coverage, vaccine hesitancy, and program implementation and evaluation. IMPACT started a working group on RSV in 2015, looking at what is available on Canadian epidemiology and planning for a surveillance project on severe outcomes in hospitalized children. These networks have found that transparent, open processes, standard operating procedures for study processes, project review and funding of have been essential in conducting research in multiple provinces across a large country.

The discussion was based on the influenza surveillance data generated by these networks, including the studies controlling for frailty in older persons. The need for significantly more data to make the right cost–effective models impressed the audience, but the overall expectation was that in a year, based on CIRN and other studies of RSV illness in the community, there will be more sophisticated models available to accurately assess this burden.

During the discussion it was emphasized what we can learn from a recent transmission dynamic cost–effectiveness model that led to the decision to extend influenza vaccination to children in the UK. A lot more data are needed to build a similar model for RSV vaccination, both in terms of better age–specific RSV virological surveillance, short– and long–term consequences of RSV infection among different age–groups, and the effect of vaccination on transmissibility of RSV. Meanwhile, cost–effectiveness models should be updated when clinical trial data and improved burden of disease estimates become available in the near future.

## DEVELOPMENT OF RSV VACCINES FOR USE IN PREGNANCY

### Clinical endpoints in trials of RSV vaccines in pregnant women: study design issues, assessment of safety and effectiveness

Maternal influenza studies were discussed as a comparator for vaccination of pregnant women against RSV. *Dr Nunes* discussed the challenges for maternal immunization especially in low– and middle–income countries (LMIC), and identified assessing the accurate gestational age as a main problem. The best method would be early ultra sound, which is not always available. Nunes emphasized the importance to know the study population for designing such a trial and gave examples of risk–factors which are more prevalent in LMIC such as co–morbidities like anemia and concomitant illnesses (HIV, malaria) that may affect the placental function. For experimental vaccines a randomized, placebo–controlled trial would be the desired study design, with a primary objective of evaluating the efficacy of RSV vaccination of pregnant women against laboratory–confirmed RSV LRTI in their infants up to 3 months of age. A major concern in maternal immunization trials is safety endpoints; in this regard the WHO requested the Brighton Collaboration (BC) to develop a guidance document harmonizing safety assessments during maternal and neonatal vaccine trials in all resource settings ie, in LIC and high IC. Although promising, maternal vaccination might be limited by transplacental antibody transfer, antibody decay rates in the infants and safety in pregnant women. Information on RSV–associated disease burden in pregnant women is lacking and will be obtained from virological analyses from the recent large maternal influenza vaccine trials.

### Lessons learned from non–RSV maternal immunization – safety, immunogenicity and effectiveness

Safety of maternal vaccination was discussed by *Dr Van der Maas*. She mentioned that based on the monitoring in a Norwegian influenza immunization study after the influenza A (H1N1) pandemic in 2009, no increased risk was found for fetal death in vaccinated women compared to non–vaccinated women, but women who contracted influenza during pregnancy did have a significantly greater risk of fetal death compared with pregnant women who did not suffer from influenza [14]. It was concluded that vaccination works and is safe, backed by other papers and a Dutch study. Follow up of the infants up to 1 year showed no difference for growth, development and GP infection–related contact rates, in infants of vaccinated and non–vaccinated mothers [15–17]. Furthermore, Van der Maas referred to the re–emergence of pertussis in the world, and emphasized the importance of monitoring the maternal pertussis vaccination for effectiveness, immunogenicity and safety. Regarding immunogenicity and the transplacental IgG transport, the timing of the vaccination is crucial. One of the lessons learned from non–RSV maternal vaccination is the essential monitoring of safety, effectiveness and immunogenicity, in order to maintain the public trust, the occurrence of disease in infants (“vaccine failure” vs “failure to vaccinate”), and to optimize the infants–, as well as the maternal vaccination schedule.

It was discussed that the most common cause of non–obstetrical fatal illness in pregnant women is in fact respiratory disease. Another point was what would be the best settings for conducting trials of such a vaccine, since the burden is highest in populations that cannot afford private health care and high cost interventions. The participants agreed that the trials should be conducted both in developed countries and in LMIC. Besides, the BMGF has partnered with industry with the aim of making these vaccines available at a lower cost in developing countries.

## CONCLUDING REMARKS

The 2016 ReSViNET meeting was organized with participation of pharmaceutical companies, public health advocates, academia, WHO, FDA, EMA and the BMGF. There was a focus on regulatory requirements for upcoming RSV therapeutics. The meeting integrated information from many of the stakeholders, including views of the regulators and public health. Alignment of the regulatory requirements with the developments in the pharmaceutical field was identified as a major challenge. Integrating the views of all stakeholders, including the patient’s perspective, will optimize the development of novel therapeutics against a respiratory virus which continues to cause so much disease to so many people worldwide.
